# Smart dosing: revolutionizing uveal melanoma treatment with AI

**DOI:** 10.1097/MS9.0000000000003669

**Published:** 2025-08-01

**Authors:** Omaima Ibrahim, Maliha Khalid, Muhammad Talha, Aminath Waafira

**Affiliations:** a Dow Medical College, Department of Medicine, Karachi, Pakistan; b Jinnah Sindh Medical University, Karachi, Pakistan; c King Edward Medical University, Lahore, Pakistan; d The Maldives National University, Malé, Maldives

**Keywords:** artificial intelligence, oncology, precision radiation therapy, radiogenomics, uveal melanoma

## Abstract

Uveal melanoma, the most common primary intraocular malignancy in adults, presents a significant challenge due to its high metastatic potential and the need to preserve vision during treatment. While conventional therapies such as plaque brachytherapy and proton beam radiation aim to balance tumor control with ocular preservation, recent advances in artificial intelligence (AI) and machine learning (ML) offer transformative potential in personalizing radiation dosing. By integrating radiologic features with genomic markers such as BAP1 mutations, monosomy 3, and chromosome 8q gain, AI models can predict tumor radiosensitivity and guide dose modulation based on individual tumor biology. This precision approach may enhance treatment efficacy while minimizing toxicity to surrounding ocular structures. However, the deployment of AI in clinical oncology also raises ethical concerns, including the risk of algorithmic bias and the need for data diversity, regulatory oversight, and interdisciplinary collaboration. Responsible integration of AI into radiation oncology could redefine treatment strategies for uveal melanoma, ushering in a new era of radiogenomics-driven precision medicine.

*Dear Editor,*

Uveal melanoma, while a rare ocular malignancy, remains the most common primary intraocular cancer in adults and carries a high risk of metastasis and mortality. Traditional management using plaque brachytherapy or proton beam radiation aims to balance tumor control with vision preservation. In 2025, we are witnessing a paradigm shift: artificial intelligence (AI) and machine learning (ML) now offer an unprecedented opportunity to differentiate radiation dosing based on radiogenomic data^[1]^.

Machine learning algorithms have been successfully useful to predict tumor response by integrating imaging biomarkers with multi-omics data, helping to classify tumors based on their biological behavior and radiation sensitivity^[2,3]^. In uveal melanoma, specific genomic alterations such as BAP1 mutations, monosomy 3, and chromosome 8q gain are known to influence prognosis and treatment resistance^[4]^. By incorporating these genetic markers with radiologic features, AI can assist in modifying radiation dose to the tumor’s biological aggressiveness, essentially improving survival while minimizing side effects.

In practical terms, AI models trained on large, multi-institutional datasets can analyze a patient’s tumor omics profile and simulate its radiation sensitivity. These predictions can help clinicians determine whether a higher dose is necessary or if alternate therapies should be considered. This approach could be particularly valuable in tumors located near critical ocular structures where dosing decisions directly impact vision preservation and quality of life^[4]^. However, the inclusion of AI into clinical oncology also introduces significant ethical and operational concerns. Real-world evidence has shown that algorithms trained on biased healthcare data can underestimate disease burden in underserved populations, as seen in algorithms that did not prioritize care for Black patients due to faulty proxies like healthcare cost^[5]^. These concerns are especially pressing in oncology, where decisions can have profound life-altering consequences.

It is essential, therefore, that AI tools undergo clinical validation, regulatory oversight, and continuous auditing to ensure safety and equity. Institutions should focus on data diversity, patient-centered transparency, and collaboration between oncologists, data scientists, and ethicists. Furthermore, educational programs should prepare future radiation oncologists to critically appraise and responsibly use AI-powered systems in clinical practice. This letter to editor is in compliance with the TITAN guideline^[6]^.

In conclusion, AI-optimized radiation dosing represents a transformative innovation in the management of uveal melanoma. With responsible integration, these technologies can elevate oncology care from generalized protocols to precision medicine. As we stand at the frontier of AI-enabled healthcare, we urge the oncologists to embrace this evolution with both enthusiasm and caution, ensuring that the benefits of technological advancement are equitably distributed and ethically governed.

## Data Availability

No data were generated for this manuscript.
